# Amino Acid Signature of Oxidative Stress in Patients with Type 2 Diabetes: Targeted Exploratory Metabolomic Research

**DOI:** 10.3390/antiox10040610

**Published:** 2021-04-15

**Authors:** Cornelia G. Bala, Adriana Rusu, Dana Ciobanu, Camelia Bucsa, Gabriela Roman

**Affiliations:** 1 Department of Diabetes and Nutrition Diseases, “Iuliu Hatieganu” University of Medicine and Pharmacy, 400006 Cluj-Napoca, Romania; cbala@umfcluj.ro (C.G.B.); dana.ciobanu@umfcluj.ro (D.C.); groman@umfcluj.ro (G.R.); 2 Drug Information Research Centre, “Iuliu Hatieganu” University of Medicine and Pharmacy, 400349 Cluj-Napoca, Romania; cfarah@umfcluj.ro

**Keywords:** nitrotyrosine, oxidative stress, amino acids, metabolomics, type 2 diabetes

## Abstract

Oxidative stress plays a key role in the development of chronic diabetes-related complications. Previous metabolomic studies showed a positive association of diabetes and insulin resistance with branched-chain amino acids (AAs) and aromatic AAs. The purpose of this research is to identify distinct metabolic changes associated with increased oxidative stress, as assessed by nitrotyrosine levels, in type 2 diabetes (T2DM). Serum samples of 80 patients with insulin-treated T2DM are analyzed by AA-targeted metabolomics using ultrahigh-performance liquid chromatography/mass spectrometry. Patients are divided into two groups based on their nitrotyrosine levels: the highest level of oxidative stress (Q4 nitrotyrosine) and lower levels (Q1–Q3 nitrotyrosine). The identification of biomarkers is performed in MetaboAnalyst version 5.0 using a t-test corrected for false discovery rate, unsupervised principal component analysis and supervised partial least-squares discriminant analysis (PLS-DA). Four AAs have significantly different levels between the groups for highest and lower oxidative stress. Cysteine, phenylalanine and tyrosine are substantially increased while citrulline is decreased (*p*-value <0.05 and variable importance in the projection [VIP] >1). Corresponding pathways that might be disrupted in patients with high oxidative stress are phenylalanine, tyrosine and tryptophan biosynthesis, arginine biosynthesis, phenylalanine metabolism, cysteine and methionine metabolism and tyrosine metabolism.

## 1. Introduction

Diabetes is a condition that is continuously rising worldwide, leading to poor health outcomes and increased healthcare costs [1]. Often, the diagnosis of type 2 diabetes mellitus (T2DM) is delayed; current evidence suggests that the diagnosis is preceded by a long hyperglycemic asymptomatic phase [2]. Increasing efforts from the scientific community have led to new evidence on early changes in diabetes that may provide a theoretical basis for early diagnosis, risk prediction and prevention strategies [3].

Oxidative stress plays an important role in diabetes complications, as well as in the development of insulin resistance [4]. This state of imbalance between the oxidative and antioxidative systems of cells and tissues results in the production of excessive free radicals as reactive oxygen or nitrogen species [5]. 3-nitrotyrosine, a biomarker of nitroxidative stress, is the main product of tyrosine oxidation by reactive nitrogen species derived from inflammatory cells, which has been identified in diabetes patients and is associated with the development of diabetes complications [6,7,8]. Nitration of tyrosine residues can profoundly alter protein structure and function, suggesting that protein nitration may be fundamentally related to and predictive of oxidative cell injury [6]. In addition to tyrosine, other amino acids (AAs) such as phenylalanine and tryptophan are modified by reactive nitrogen species [6].

The metabolomics biological technique can simultaneously analyze multiple metabolites in biological systems, such as blood and urine, through nuclear magnetic resonance chromatography and mass spectrometry (MS) [9]. In targeted metabolomics, absolute concentrations of a limited number of known metabolites are quantified, giving this method high sensitivity and selectivity [10]. In T2DM, metabolomics has led to the discovery of several metabolic biomarkers closely related to the occurrence, development and progress of the disease [11]. Various regulatory pathways, including the regulation of sugar, fat and protein are found to be disturbed in T2DM [3]. Within the protein pathway, the levels of branched-chain AAs (isoleucine, leucine, valine) and aromatic AAs (tyrosine, phenylalanine, tryptophan) are increased in T2DM patients, as found in both prospective and non-prospective studies [3]. Several studies have shown that the level of branched-chain AA can be up to 1.5-fold or even 2-fold higher in T2DM patients, as compared to healthy subjects [12]. Additionally, the plasma levels of branched-chain AAs and aromatic AAs may also predict the risk of developing T2DM in healthy individuals [9].

This analysis presented here is part of an observational cross-sectional study that aims to investigate the hypothesis that chronic administration of higher insulin doses in patients with T2DM is associated with inflammation, oxidative stress and endothelial dysfunction [13]. The objective of the analysis presented here is to identify specific changes in AA concentrations as biomarkers of increased oxidative stress, as assessed by the nitrotyrosine level, in patients with late-stage, insulin-treated T2DM.

## 2. Materials and Methods

### 2.1. Study Design and Participants

As previously described [13], this observational cross-sectional study was performed in the Diabetes and Nutrition Diseases Center at Emergency County Clinical Hospital Cluj-Napoca, Romania (trial registration ACTRN12616001542482). We enrolled 80 consecutive adult patients with T2DM, all of whom had been treated with insulin (monotherapy or as an add-on to other hypoglycemic agents) for at least six months, and with a stable insulin dose for the three months before their study inclusion, and who were presenting for a regular appointment between July and November 2017. We excluded patients with type 1 and other specific types of diabetes—including gestational diabetes; diabetic ketoacidosis and hyperglycemic-hyperosmolar state— along with those with acute illnesses able to influence the current insulin doses, acute infections, alanine aminotransferase (ALAT) and/or aspartate aminotransferase (ASAT) >3 times the upper normal limit, estimated glomerular filtration rate <30 mL/min/1.73 m^2^, those who had taken anti-inflammatory drugs (except aspirin <300 mg/day) within the previous three months, and any who were pregnant and/or lactating. We chose to include only insulin-treated patients to obtain a more homogenous group of T2DM patients in terms of diabetes duration and treatment, and focusing on those with the highest risk of chronic complications associated with exposure to hyperglycemia and oxidative stress. However, given that insulin treatment as monotherapy is rarely used in T2DM (only during pregnancy or when severe comorbidities preclude the use of other antihyperglycemic agents), we included consecutive patients treated with insulin in association with any other antihyperglycemic drugs.

The study protocol, information sheet and informed consent form were approved by the Ethics Committee of the “Iuliu Hatieganu” University of Medicine and Pharmacy Cluj-Napoca, Romania. The study was conducted according to the International Conference on Harmonization’s Good Clinical Practice Guidelines and the Declaration of Helsinki. All patients were informed of the nature of the research and personal data collection and provided written informed consent before any study-related procedure.

### 2.2. Study Assessments and Data Collection

The study visit occurred in the morning, in fasting conditions. We collected data on age, gender and medical history (diabetes duration and therapy, concomitant diseases and therapy, number of hypoglycemic episodes within 30 days prior to study enrollment) by means of patient interviews, clinical examination and from their medical files. The presence of chronic diabetes complications (diabetic retinopathy and diabetic peripheral neuropathy) was assessed by eye fundoscopy and foot exam. Height, weight and waist circumference were measured with patients wearing light clothes and no shoes. Blood pressure was measured in a sitting position after 5 min of rest.

Blood samples were collected, also in fasting conditions, and were used for the assessment of biochemical parameters (blood glucose, glycated hemoglobin A1c [HbA1c], creatinine, total cholesterol, LDL and HDL cholesterol, triglycerides, ASAT, ALAT, nitrotyrosine) and serum AA levels. Biochemical measurements were performed on the day of collection using routine enzymatic methods. Samples for the nitrotyrosine and AA were centrifuged on the same day and frozen until assessment. Nitrotyrosine levels were assessed using a commercially available enzyme-linked immunosorbent assay (ELISA) sandwich test, according to manufacturers’ instructions (Hycult Biotech, USA) [13]. An estimated glomerular filtration rate was calculated using the Chronic Kidney Disease Epidemiology Collaboration formula, available online at: https://qxmd.com/calculate/calculator_251/egfr-using-ckd-epi (last accessed 16 February 2021).

### 2.3. Serum AA Levels Measurement

The AA profile and quantification of all serum samples were determined by ultrahigh performance liquid chromatography (UHPLC) coupled with electrospray ionization-quadrupole-time of MS in positive mode (UHPLC-Q-TOF –[ESI+]-MS), using the Thermo ScientificTM UHPLC UltiMate 3000 equipped with a quaternary pump Dionex delivery system (Thermo Fisher Scientific, USA) and MS detection with the MaXis Impact 2012 version (Bruker Daltonics, MA, USA). The UHPLC-Q-TOF-(ESI+)-MS analysis and data processing of AA levels were conducted at the Research and Development Center for Applied Biotechnology in Diagnosis and Molecular Therapies, Cluj-Napoca, Romania.

The solvents used for the UHPLC analysis were acetonitrile (Merck, Germany), sodium and ammonium formate (99% purity) (Alfa Aesar, Germany) and HClO_4_ (Merck, Germany). Proteins from the serum samples were precipitated using 0.4N HClO_4_ in a 1:1 ratio, vortexed for 20 s and centrifuged at 12,500 rpm for 5 min. The supernatant was collected, filtered through a 0.2 μm Nylon filter and injected into the HPLC-MS system.

Twenty AAs were simultaneously identified: essential (histidine, isoleucine, leucine, lysine, methionine, phenylalanine, threonine, tryptophan, valine), conditional essential (asparagine, glutamine, proline, tyrosine, cysteine, arginine) and non-essential (aspartic acid, cysteine, citrulline, glutamic acid, ornithine). AAs were quantified using signal areas at retention times specific to each amino acid, using QuantAnalysis 2.2 software (Bruker Daltonics, Germany).

The quantification and absolute linearity of the 20 AAs were performed using a standard solution containing the 20 AAs in equal amounts. The standard AA solution was prepared according to the protocol used for the biofluids described above. Three-point calibration curves were performed for each individual AA quantification. Homoarginine, methionine-D3 and homophenylalanine were used as internal standards.

The free AAs obtained from the derivatization were separated on the Intrada Amino Acids 50 × 3 mm, 3 µm (Imtakt, Kyoto, Japan). The mobile phases consisted of acetonitrile/formic acid (100/0.3) and acetonitrile/ammonium formate 100 mM (20/80). The flow rate was set at 0.6 mL/min and the column temperature was maintained at 37 °C, while the elution time was set at 24 min and the injected volume was 5 μL. The identification of derivatized AAs was done with MS using the following parameters: the ionization electrospray source was set in the positive ion mode, the nebulization gas pressure was 2.8 bars, the dry gas flow was 12 L/min and the drying gas temperature was set at 300 °C. Prior to each injection, a sodium formate calibration solution was introduced into the system. Instrument control and data processing were performed using a number of specific software: TofControl 3.2, HyStar 3.2, Data Analysis 4.2 and Quantitative Analysis 2.2 (Bruker Daltonics, MA, USA).

### 2.4. Statistical Analysis

Participants were divided into two groups based on their nitrotyrosine levels: the highest level of oxidative stress (quartile 4 [Q4] nitrotyrosine) and lower levels (Q1–Q3 nitrotyrosine). Anthropometric and clinical variables were described using means ± standard deviation and median (Q1; Q3) for continuous variables and number (percentage) for discrete variables. Nitrotyrosine groups were compared for differences in these variables by student t, median and chi-square tests using SPSS version 26 (IBM Corp., USA). Statistical analysis of metabolomic parameters was performed in MetaboAnalyst version 5.0. Metabolomic data were log transformed for normalization. Oxidative stress groups were compared using univariate analysis methods (t-tests). Metabolites with a false discovery rate (FDR) corrected *p*-value <0.05 were considered statistically different between groups. Unsupervised principal component analysis (PCA) and supervised partial least-squares discriminant analysis (PLS-DA) were employed to identify intrinsic variation in the studied metabolites and to screen for potential biomarkers. Particularly, PLS-DA was applied to model metabolites’ variations in the two groups, and the best-fitted model was used to assess the variable importance in the projection (VIP) for all metabolites. To account for the low sample size, especially in the Q4 nitrotyrosine group, we used cross-validation and permutation, which were performed as part of the PLS-DA procedure, to validate the models. The goodness of fit of the PLS-DA model was determined by internal cross-validation testing (10-fold CV) and calculation of R2 (for the explanation of the total variations in the data) and Q2 (to provide an estimate of the predictive ability of the models). To improve data visualization, a heatmap was generated using metabolites and samples, by employing Euclidean for distance measures and Ward for the clustering algorithm. Finally, metabolites with VIP values of greater than 1 and an FDR *p*-value in t-test of less than 0.05 were selected for pathway analysis, which was also performed in MetaboAnalyst version 5.0.

## 3. Results

### 3.1. Participants’ Characteristics

As stated previously [13], 80 patients with T2DM treated with insulin were included in this research. The study group included mostly women, with a mean BMI of 32.0 kg/m^2^ in the Q4 tyrosine group and 32.7 kg/m^2^ in the Q1–Q3 tyrosine group. The mean diabetes duration was 14.5 years in the Q4 tyrosine group and 12.3 years in the Q1–Q3 tyrosine group. The most frequent diabetes complication was diabetic peripheral neuropathy in both groups and most patients had high blood pressure and dyslipidemia. No difference in the median number of hypoglycemic episodes within 30 days prior to study enrollment was observed between the study groups. Nitrotyrosine levels were 66.5 nmol/mL in the Q4 tyrosine group and 22.9 nmol/mL in the Q1–Q3 tyrosine group (Table 1).

### 3.2. Metabolites’ Identification

Targeted metabolomics of the serum samples was applied to identify metabolites that were different in the groups with the highest and lower levels of oxidative stress. By t-test analysis with FDR correction, three essential AAs, three conditional essential AAs and one non-essential AA were found to be statistically significantly different between the groups. Essential (phenylalanine, isoleucine, methionine) and conditional essential (tyrosine, cysteine, proline) AAs were significantly higher in patients in Q4 of nitrotyrosine, and citrulline (non-essential AA) was significantly lower in this group when compared to patients in Q1–Q3 of nitrotyrosine (Table 2).

**Figure 1:**
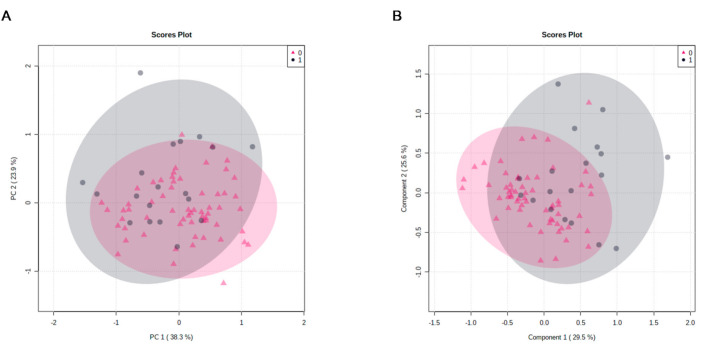
PCA (panel **A**) and PLS-DA (panel **B**) component analysis score plot between the selected principal components. PCA, principal component analysis; PLS-DA, partial least-squares discriminant analysis; 0 refers to lower oxidative stress (Q1–Q3 nitrotyrosine) group (pink triangle); 1 refers to highest oxidative stress (Q4 nitrotyrosine) group (grey circles).

Unsupervised PCA was further employed to display the general trend of AAs’ concentrations and to identify potential outliers in the analyzed samples. Using the PCA method, we identified five factors that, overall, explained 87.6% of the variance, showing satisfactory clustering between the Q4 and Q1–Q3 nitrotyrosine groups, as displayed in the PCA score plot examples provided in Figure 1A.

**Figure 2:**
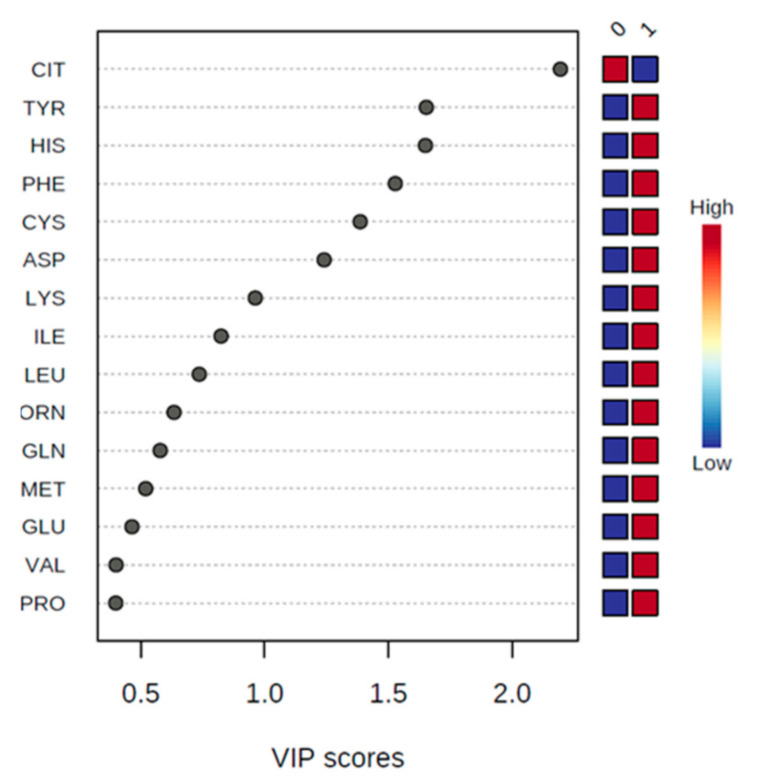
Important metabolites identified by PLS-DA. The colored boxes on the right indicate the relative concentrations of the corresponding metabolite in each group under study. The lower oxidative stress (Q1–Q3 nitrotyrosine) group is referred to by 0; the highest oxidative stress (Q4 nitrotyrosine) group’s PLS-DA (partial least-squares discriminant analysis) is referred to by 1; VIP, variable importance in the projection.

Supervised PLS-DA was further performed to search for discriminating AAs between the groups with the highest and lower levels of oxidative stress. The best PLSA-DA model we obtained had five components, explaining 29.5%, 25.6%, 15.9%, 4.1% and 7.6% of the variation, respectively. In cross-validation, the accuracy of this model was 0.78 and R^2^ and Q^2^ were 0.46 and 0.17, respectively. A partial separation between the two groups was also observed in the PLSA-DA score plots, showing good description but weak prediction (Figure 1B). These results were confirmed by the permutation test, which showed an empirical *p*-value of 0.02 for prediction accuracy during training and separation distance (100 permutations performed). The PLS-DA model was further analyzed by VIP scores to identify the AAs contributing to sample discrimination. Six metabolites with VIP scores >1 and thus with significant variation between groups were identified: asparagine (VIP predicted value: 1.2), cysteine (VIP predicted value: 1.4), phenylalanine (VIP predicted value: 1.5), histidine (VIP predicted value: 1.6), tyrosine (VIP predicted value: 1.7) and citrulline (VIP predicted value: 2.2) (Figure 2). Metabolites further away from the origin made higher contributions to the separation between the Q4 and Q1–Q3 nitrotyrosine groups.

**Figure 3:**
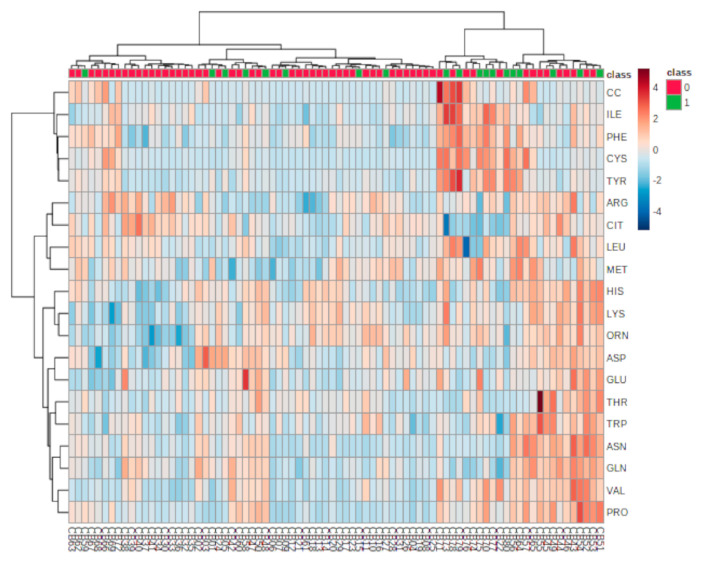
Heatmap of targeted metabolomic analysis for the correlation between analyzed amino acids and individual samples. Rows, amino acids; columns, individual samples. Color key shows the metabolite expression value: lowest (blue) and highest (red); 0 refers to lower oxidative stress (Q1–Q3 nitrotyrosine) group; 1 refers to highest oxidative stress (Q4 nitrotyrosine) group.

The results of the hierarchical clustering analysis are displayed as a heatmap in Figure 3, presented as a visual aid for the correlation between the concentrations of analyzed AAs in individual samples grouped into lower oxidative stress (Q1–Q3 nitrotyrosine) or highest oxidative stress (Q4 nitrotyrosine).

### 3.3. Metabolic Analysis Pathway

**Figure 4:**
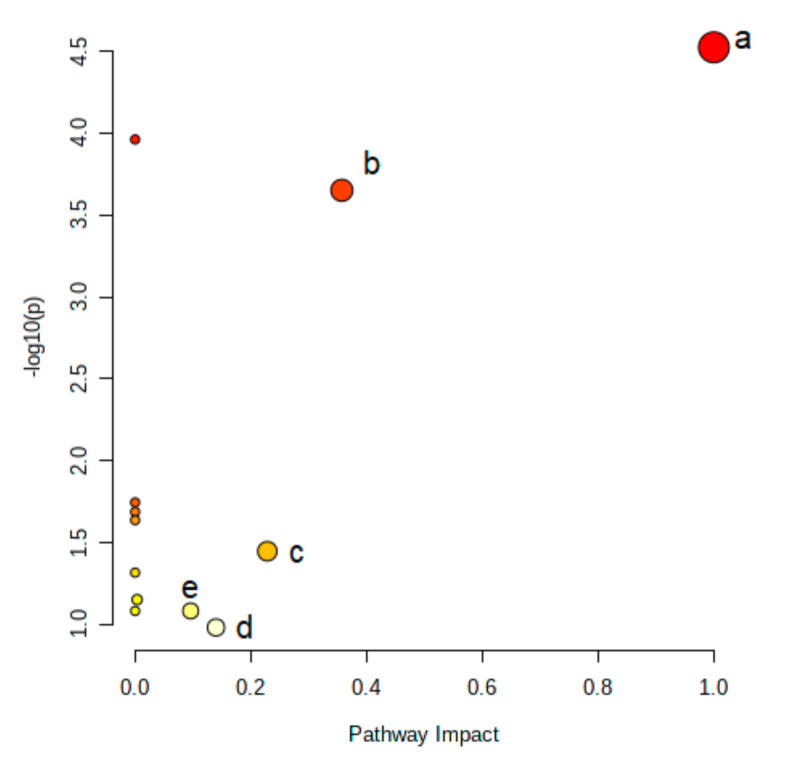
Summary of pathway analysis in patients with highest vs. lower oxidative stress.

Finally, based on FDR-corrected *p*-values for the AAs listed in Table 1 and the VIP scores listed in Figure 2, four AAs were selected for pathway analysis that each had a *p*-value <0.05 and a VIP score >1 and which were considered as best reflecting the metabolic differences between Q4 and Q1–Q3 of nitrotyrosine: cysteine, phenylalanine, tyrosine and citrulline. The pathway analysis combined results from a powerful pathway enrichment analysis with a pathway topology analysis to identify the most relevant pathways involved in the conditions under study, according to the selected group of metabolites in the previous steps. Based on a pathway impact value of >0, five target pathways were identified as altered in patients in Q4 of nitrotyrosine: phenylalanine, tyrosine and tryptophan biosynthesis, phenylalanine metabolism, arginine biosynthesis, cysteine and methionine metabolism and tyrosine metabolism, with pathway impact values of 1.00, 0.36, 0.23, 0.14 and 0.10, respectively (Figure 4).

A—phenylalanine, tyrosine and tryptophan biosynthesis; b—phenylalanine metabolism; c—arginine biosynthesis; d—tyrosine metabolism; e—cysteine and methionine metabolism.

## 4. Discussion

Using a targeted metabolomic approach, in this analysis, we aimed to identify metabolites associated with high oxidative stress in T2DM. We identified four metabolites (AAs) associated with the highest level of nitrotyrosine: cysteine, phenylalanine, tyrosine (significantly higher) and citrulline (significantly lower). The identified AAs may be linked to the level of oxidative stress in patients with T2DM. To the best of our knowledge, no previous research has examined metabolite AAs associated with oxidative stress in diabetes, although a large amount of data on biomarkers, including other metabolites and diabetes complications, are available.

Cysteine, a proteinogenic non-essential sulfur-containing AA, can be sourced from the diet or produced via methionine degradation [14]. In the human body, cysteine is involved in lipid, keratin and iron–sulfur (a constituent of skeletal muscle) biosynthesis and represents a precursor of the antioxidant glutathione and of the coenzyme A, which is involved in cellular oxidative stress [14]. Higher serum cysteine levels have been reported in metabolic syndrome and obesity [15], and have been hypothesized to have an insulin-like action at the adipocyte level [16]. Furthermore, the cysteine level has been positively correlated with total fat mass [17], insulin resistance, inflammation [15] and endothelial dysfunction [18]. Also, vascular toxicity of cysteine has been described: the autoxidation of cysteine generates reactive oxygen species (ROS) that can alter LDL-cholesterol [19] and decrease endothelium-dependent vasodilation by O_2_ generation [20]. Thus, higher cysteine levels are associated with an altered plasma redox state [21], which was demonstrated in our subjects with late-stage, insulin-treated T2DM.

Increased levels of aromatic AAs (phenylalanine and tyrosine) have been associated in follow-up studies with the development of insulin resistance [22], decreased insulin secretion [23] and an increased risk of developing T2DM, suggesting a role of these AAs in the pathogenesis of diabetes [24]. In previous prospective research, phenylalanine levels were positively associated with increased risk of mortality among patients with T2DM, although the association was not independent [25]. The results of research on the association of tyrosine levels with the risk of diabetic complications have been mixed, with some showing a positive association and several showing an inverse association. In a case-cohort analysis of the data from the Action in Diabetes and Vascular Disease: Preterax and Diamicron MR Controlled Evaluation (ADVANCE) trial, enrolling 3587 patients with T2DM, higher tyrosine levels at the baseline were associated with a lower risk of developing microvascular complications (HR 0.78 [95% CI: 0.67–0.91]) [25]. Conversely, in a study comparing patients with diabetic nephropathy, T2DM without nephropathy and healthy subjects, higher plasma tyrosine levels were associated with higher odds for diabetic nephropathy (OR 0.329 [95% CI: 0.144–0.750]) after adjustment for age, sex, BMI, blood pressure, lipid levels and antihypertensive medication use [26]. The higher phenylalanine levels observed in the group with the highest oxidative stress in our sample may be explained by the effect of oxidative stress on tyrosine metabolism. Tyrosine can be synthesized from phenylalanine by phenylalanine hydroxylase at the kidney and liver level [27]. Increased oxidative stress limits the availability of tetrahydrobiopterin, a cofactor of phenylalanine hydroxylase, with consequent impairment of phenylalanine hydroxylase, a decreased conversion rate of phenylalanine to tyrosine and increased phenylalanine levels [28]. Additionally, increased oxidative stress is associated with increased formation of nitrotyrosine from phenylalanine and tyrosine [29,30] and thus may explain the high levels of these aromatic AAs observed in our group in the highest nitrotyrosine quartile.

Citrulline is a non-essential AA, resulting as an intermediate product of the urea cycle and as a byproduct of nitric oxide (NO) formation by endothelial nitric oxide synthase (eNOS) in the presence of (6R)-5,6,7,8-tetrahydro-l-biopterin (BH4) as a cofactor [31]. One of the central mechanisms of macrovascular complication in T2DM is endothelial dysfunction, involved not only in the initiation and progression of atherosclerosis, but also in the transition from a stable to an unstable disease state [32]. It precedes the development of morphological atherosclerotic changes and may also contribute to lesion progression and later to rupture or erosion, and trigger thrombogenic phenomena [32,33,34]. NO is a key mediator in the regulation of vascular tone, platelet aggregation and vascular smooth cell proliferation, and thus, reduced NO levels are contributing factors to endothelial dysfunction in diabetes [35]. T2DM is associated with increased ROS production. In turn, ROS reacts with vascular NO, resulting in peroxynitrite, which further oxidizes the cofactor BH4 and determines eNOS dysfunction/uncoupling, with consequent NO and citrulline production [35]. This may explain the lower levels of citrulline observed in the highest oxidative stress group in our research.

Hyperglycemia-associated oxidative stress has been identified as a key contributor to endothelial dysfunction and vascular inflammation in T2DM, supporting its central role in the development of chronic diabetes complications [36]. In our study, although the frequency of diabetic neuropathy and retinopathy was numerically higher in the group with higher oxidative stress than in that with lower oxidative stress, the difference did not reach statistical significance, most probably due to the small sample size. Our study was a cross-sectional one; by design, these studies cannot infer causality and are not able to capture long-term exposure to factors known to contribute to chronic complications. Also, it has previously been shown that acute hyperglycemia is associated with an acute increase in nitrotyrosine levels [37]. However, in patients with T2DM, nitrotyrosine levels are associated with glycemic variability [38,39] and not with HbA1c values [7]. Similarly, in our study, we found no differences in HbA1c values between the groups with the highest and lower oxidative stress. Although our work is the first one investigating AAs biomarkers of oxidative stress in T2DM using metabolomic techniques, it has several limitations that we must acknowledge. First, it is a cross-sectional study and thus it does not demonstrate a causal relationship of oxidative stress with the identified AAs. Another limitation is the small sample size, which may explain the partial separation of the groups in the PCA and PLSA-DA models. As a result, the Q4 nitrotyrosine group was also small, but the use of PCA and PLSA-DA methods with cross-validation and permutation overcame the small number of AA samples. Also, we only enrolled patients treated with insulin. While this limited the heterogeneity of our sample, it may have also influenced the identified biomarkers. Another limitation is the imbalance in the add-on therapy to insulin in the two groups, which resulted from the consecutive enrollment of the patients in the research. The percentage of patients treated with GLP-1 receptor agonists and sulfonylurea was significantly higher in the highest oxidative stress (Q4 nitrotyrosine) group than in the lower oxidative stress (Q1–Q3 nitrotyrosine) group. Previous research has shown that therapy with GLP-1 receptor agonists is associated with reductions in markers of oxidative stress [40]. Sulfonylurea gliclazide also has antioxidant activities, while other sulfonylurea shows no such activities [41,42,43]. Thus, the imbalance noted in the therapy with these drugs with higher use in patients with higher oxidative stress may have had only minor effects on the separation of the models identified during the statistical analysis, but further controlling for these drugs may improve the performance of identified models.

## 5. Conclusions

In summary, by targeting AAs’ metabolomics, we identified four biomarkers associated with increased oxidative stress in insulin-treated T2DM. Our results indicate that high oxidative stress is associated with higher cysteine, phenylalanine and tyrosine and with lower citrulline levels. Corresponding pathways that might be disrupted in patients with high oxidative stress are phenylalanine, tyrosine and tryptophan biosynthesis, arginine biosynthesis, phenylalanine metabolism, cysteine and methionine metabolism and tyrosine metabolism. Further research on a larger number of individuals with T2DM, with the sample followed prospectively, is needed to confirm if alterations to AAs and their corresponding pathways may play a role in the development of chronic complications via oxidative stress, and might be used as drug targets against the progression of diabetes complications.

## Figures and Tables

**Table 1:** Participants’ characteristics according to nitrotyrosine quartiles.

	Q4 Nitrotyrosine N = 19	Q1–Q3 Nitrotyrosine N = 61	*p*-Value
Women, n (%)	13 (68.4%)	34 (55.7%)	0.427
Smoking, n (%)	1 (5.3%)	8 (13.1%)	0.678
BMI, kg/m^2^	32.0 ± 4.6	32.7 ± 6.0	0.668
Diabetes duration, years	14.5 ± 7.7	12.3 ± 6.8	0.251
FPG, mg/dL	187.9 ± 62.1	177.5 ± 71.2	0.567
HbA1c, %	8.1 ± 1.2	8.5 ± 1.7	0.301
LDL cholesterol, mg/dL	86.6 ± 39.7	95.7 ± 34.9	0.340
HDL cholesterol, mg/dL	45.1 ± 11.6	46.7 ± 11.0	0.580
Triglycerides, mg/dL	196.6 ± 98.9	174.5 ± 78.5	0.319
Diabetic neuropathy, n (%)	15 (78.9%)	36 (59.0%)	0.172
Diabetic retinopathy, n (%)	6 (31.6%)	16 (26.2%)	0.651
Diabetic kidney disease, n (%)	7 (36.8%)	30 (49.2%)	0.148
HBP, n (%)	15 (78.9%)	54 (88.5%)	0.281
DLP, n (%)	15 (78.9%)	48 (78.7%)	1.00
CVD, n (%)	8 (42.1%)	25 (41.0%)	1.00
Diabetes therapy, n (%)			
Insulin	19 (100.0%)	61 (100.0%)	-
Metformin	14 (73.7%)	39 (63.9%)	0.581
Sulfonylurea	3 (15.8%)	1 (1.6%)	0.040
GLP1 RA	4 (21.1%)	2 (3.3%)	0.026
DPP-4i	0 (0.0%)	1 (1.6%)	1.00
α-glucosidase inhibitor	0 (0.0%)	2 (3.3%)	1.00
Thiazolidinediones	1 (5.3%)	0 (0.0%)	0.237
Number of hypoglycemia in the previous 30 days	0 (0; 1)	0 (0; 1)	0.971
Nitrotyrosine, nmol/ml	66.5 (48.5; 96.0)	22.9 (19.1; 26.7)	<0.001

N/n (%), number (percentage) of participants; Q, quartile; BMI, body mass index; FPG, fasting blood glucose; HbA1c, glycated hemoglobin; HBP, high blood pressure; DLP, dyslipidemia; CVD, cardiovascular disease; GLP1 RA, glucagon-like peptide-1 receptor agonist; DPP-4i, dipeptidyl-peptidase-4 inhibitor.

**Table 2:** Metabolites with significant differences between groups, according to a t-test.

	Q4 Nitrotyrosine N = 19	Q1–Q3 Nitrotyrosine N = 61	*p*-Value	FDR Corrected *p*-Value
Tyrosine	45.1 (39.4; 116.7)	36.0 (33.2; 41.6)	1.7693e-05	0.00035385
Phenylalanine	89.2 (64.3; 150.5)	59.3 (44.7; 77.9)	0.00025327	0.0025327
Cysteine	84.7 (69.7; 213.0)	67.7 (65.3; 78.7)	0.0013475	0.0089834
Citrulline	11.5 (4.9; 14.8)	17.0 (9.7; 25.5)	0.0040269	0.020134
Isoleucine	46.3 (40.5; 69.4)	40.8 (37.7; 47.0)	0.0068384	0.027354
Proline	163.3 (140.7; 179.4)	139.0 (130.8; 157.4)	0.0083898	0.027966
Methionine	13.1 (11.7; 15.4)	11.9 (10.2; 13.6)	0.012433	0.035522

N/n (%), number (percentage) of participants; Q, quartile; FDR, false discovery rates.

## Data Availability

The data presented in this study are available on request from the corresponding author. The data are not publicly available as written consent was not obtained from study participants for this.
